# Contamination detection by optical measurements in a real‐life environment: A hospital case study

**DOI:** 10.1002/jbio.201960069

**Published:** 2019-11-06

**Authors:** Jenni Inkinen, Merja Ahonen, Evgenia Iakovleva, Pasi Karppinen, Eelis Mielonen, Riika Mäkinen, Katriina Mannonen, Juha Koivisto

**Affiliations:** ^1^ Aalto University, School of Science, Department of Applied Physics Complex Systems and Materials Aalto Finland; ^2^ Satakunta University of Applied Sciences, Faculty of Technology WANDER Nordic Water and Materials Institute Rauma Finland

**Keywords:** environmental monitoring, health‐care associated infections, hyperspectral, infection control, optical imaging

## Abstract

Organic dirt on touch surfaces can be biological contaminants (microbes) or nutrients for those but is often invisible by the human eye causing challenges for evaluating the need for cleaning. Using hyperspectral scanning algorithm, touch surface cleanliness monitoring by optical imaging was studied in a real‐life hospital environment. As the highlight, a human eye invisible stain from a dirty chair armrest was revealed manually with algorithms including threshold levels for intensity and clustering analysis with two excitation lights (green and red) and one bandpass filter (wavelength λ = 500 nm). The same result was confirmed by automatic k‐means clustering analysis from the entire dirty data of visible light (red, green and blue) and filters 420 to 720 nm with 20 nm increments. Overall, the collected touch surface samples (N = 156) indicated the need for cleaning in some locations by the high culturable bacteria and adenosine triphosphate counts despite the lack of visible dirt. Examples of such locations were toilet door lock knobs and busy registration desk armchairs. Thus, the studied optical imaging system utilizing the safe visible light area shows a promising method for touch surface cleanliness evaluation in real‐life environments.
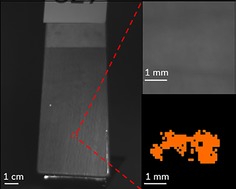

## Graphical abstract

Touch surface cleanliness monitoring by optical imaging was studied in a real‐life hospital environment using both manual and automatic hyperspectral scanning algorithms. As the highlight, a human eye invisible stain from a dirty chair armrest was revealed. Thus, the optical imaging system in the safe visible light area shows a promising method for touch surface cleanliness evaluation in real‐life environments.

## 1 INTRODUCTION

Touch surfaces are an important source of bacteria and many pathogenic and opportunistic bacteria can persist on a surface even for months, these contaminated surfaces can further contribute to transmission of pathogens that can even cause hospital acquired infections 1, 2. The important biological contaminants vary depending on the environment; in the hospital environment, many antibiotic‐resistant strains nowadays pose challenges such as methicillin‐resistant *Staphylococcus aureus*, vancomycin‐resistant *Enterococcus* and *Clostridium difficile* 1, 3. Despite these challenges, a real‐time monitoring system is still lacking to indicate biological contamination on touch surfaces and current methods include fast but unspecific adenosine triphosphate (ATP) measurement and slow cultivation techniques. Despite its rapidity, commercial ATP swab tests provide an estimate of total organic soil (food residues and microbial cells) 4 and thus are unable to differentiate whether the contamination is microorganism based or, for example, organic food. A limiting factor in traditional microbiological cultivation techniques in addition to long cultivation time is the occurrence of viable but nonculturable bacteria that might underestimate real microbial load in touch surfaces 5. Given that infectious doses can be extremely low, for example, less than 10 spores or colony‐forming units (CFU), the detection limit of bacteria is crucial in the infection control in hospital environment 6. Analyzing also other nonbiological organic materials is relevant as they may pose potential nutrients for the bacteria enabling bacterial growth.

Visual inspection of a surface is not equal to microbiological swabbing or ATP test to indicate unsatisfactory touch surface cleanliness, and therefore cannot predict the risk of microbial transmission from contaminated surfaces 7, 8. The biological contamination detection even in real time is possible using different intrinsic fluorescence properties of the biological contaminants and has been mainly studied in food industry applications 9, 10, 11 or monitoring the cleaning efficiency in food processing facilities 12. Hyperspectral imaging has shown potential in biomedical applications, for example, to distinguish tumorous and normal tissues, detect changes in blood cells or mucosal surfaces, or to detect living microorganisms such as *Amoeba proteus* 13, 14, 15, 16, 17. In principle, depending on a target compound of the electromagnetic radiation, different fluorescence emissions are detected from a microorganism or food components. At excitation wavelengths of UV light, intracellular nucleic acids (DNA and RNA) and amino acids such as tyrosine and phenylalanine but especially tryptophan that is present in a cell wall of the bacteria emit light in the UV to blue light range 18, 19, 20, 21. The presence of the nucleic acids and amino acids as mentioned above are not dependent on the metabolic state of the cell, for example, these are found in the cell until cell death whereas intracellular coenzymes (NADH, flavins) can give more details about metabolic state of the cell such as growth conditions 18. In visible excitation wavelength of blue light, flavins especially riboflavin (vitamin B2) emit green light 18. Many studies of bacteria present in varying food products have shown absorbance and/or reflectance in the higher electromagnetic radiation wavelengths 10. Only one study has utilized hyperspectral imaging to detect clinical contamination in a hospital 21. In this study, tryptophan was suggested as the most reliable fluorophore over, for example, cellular metabolites such as NADH 21. Surfaces electromagnetic spectrum is often analyzed with chemometrics, for example, principal component analysis or artificial neural network that are machine learning‐based algorithms designed to detect and identify possible surface contaminations where every contamination has its own “fingerprint” in electromagnetic spectrum 11. Utilizing chemometrics and/or the intrinsic fluorescence spectra of microorganisms, even different bacterial or fungal strains can be separated from each other 22, 23, 24. Further, live bacteria yield stronger fluorescence signal in most sensitive measurements can differentiate live and dead bacteria 25.

Differentiating Gram‐negative *Escherichia coli* and Gram‐positive *Bacillus subtilis* from the background and from each other with varying concentrations was performed in laboratory by hyperspectral imaging 24. The aim of this study was to study hyperspectral imaging further in real‐life environment and to confirm the usability of this system (AutoDet) for detecting visible and invisible stains to the human eye on touch surfaces in a hospital. Both manual algorithms/methods as well as machine vision algorithms were used.

## 2 METHODS

### 2.1 Studied touch surfaces and sampling sites

**Figure 1:**
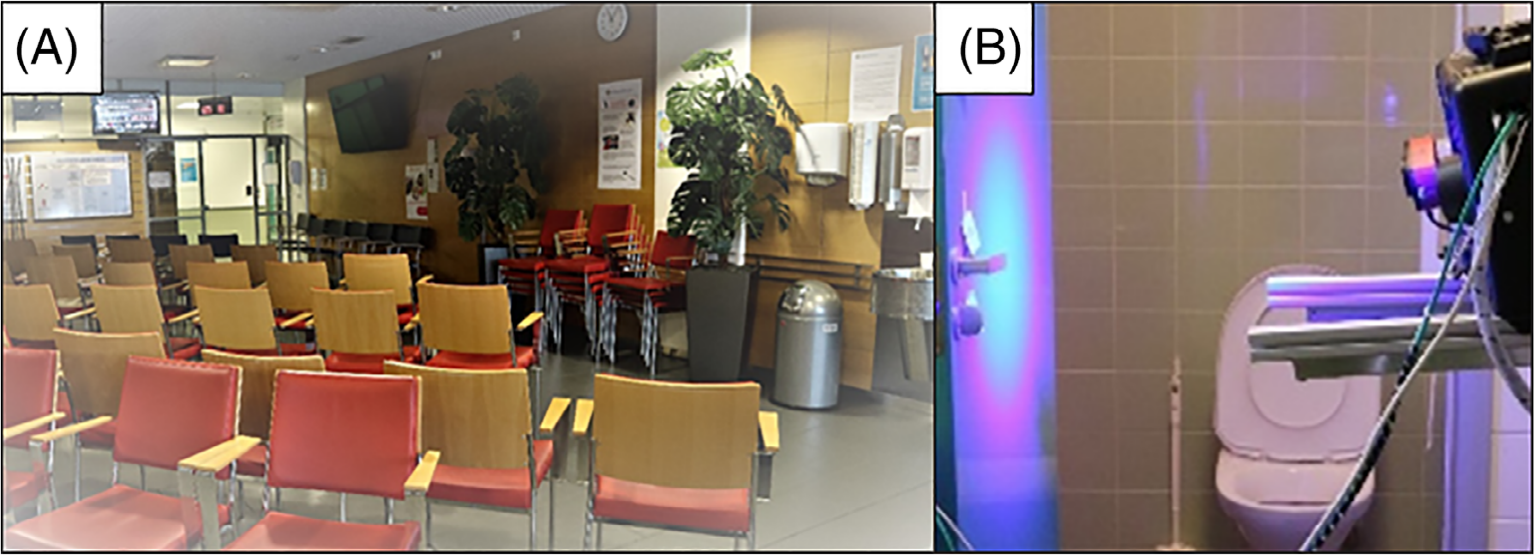
Studied real‐life hospital environment, that is, (A) an emergency waiting area and (B) a toilet where the optical device is imaging a studied door handle

Real‐life touch surface sampling was performed in a hospital located in Finland (Figure 1). Touch surfaces from common lounge areas and toilets as listed in Table 1, including traditional products without antimicrobial properties as well as commercially available products with antimicrobial silver‐based coatings. During March to April 2019, 156 samples were collected in five sampling days on Mondays at prior first cleaning of the week that represented presumably the worst‐case scenario by surface dirtiness. From each surface, either culturable bacteria (total plate count, TPC) or microbial biomass and organic dirt (ie, ATP) was analyzed. In addition, 66 samples were collected for optical measurements (AutoDet). For these ATP and optical measurements, the similar images after cleaning (with a wet microfiber cloth and 80% ethanol solution) were compared to dirty samples to confirm the occurrence of dirt. In the hospital, toilets were routinely cleaned daily by cleaning professionals with nondisinfecting and nonionic detergent (Yleispesu Joutsen, Berner, Finland) and once per week on Mondays with alkaline detergent especially designed for greasy dirt (C4 max, KiiltoClean Oy, Finland). The emergency room personnel cleaned emergency room common areas during the weekends with nondisinfecting and nonionic detergent (Yleispesu Joutsen, Berner, Finland). In all cases of secretions, chlorine‐based disinfecting detergent was used for cleaning.

**Table 1:** Experimental design from real‐life hospital touch surfaces and overall hygienic levels between different locations

		ATP RLU/cm^2^					TPC CFU/cm^2^ and indicator bacteria							
Sampling location	Surface area (cm^2^)	Average (ATP)	SD (ATP)	Min (ATP)	Max (ATP)	N (ATP)	Average (TPC)	SD (TPC)	Min (TPC)	Max (TPC)	N (TPC, indicators)	*S. aureus* a (%)	*Enterococci* a (%)	Gram‐negatives^a^ (%)
Common area table	100	1.2	1.2	0.1	3.9	11	3.6	7.1	0.5	29	14	7.1	7.1	0
Chair armrest emergeny unit	47–53	6.0	5.0	0.1	16	16	7.8	16.6	0.5	85	24	8.3	0.0	0
Chair armrest childrens' unit	44					‐	6.4	7.5	0.5	37	34	2.9	8.8	0
Toilet door handle	23–25	1.4	1.9	0.0	6.0	9	6.2	8.6	0.5	7.0	6	33.3	16.7	0
Toilet door puller	64	4.9	4.5	0.2	14	8	1.3	0.8	0.5	3.0	10	20	0	0
Toilet door lock	37					‐	21.5	48.4	1.0	190	14	35.7	7	0
Total		3.6	4.3	0.0	16	44	7.8	20.4	0.5	190	112	11.6	5	0
Statistical significance^b^ *P* value (sampling location)		*P* < .05					*P* < .05, post hoc: toilet door lock vs toilet door puller *P* < .007							

a Percentage of positive findings per total number of samples.

b Statistical significance level of ATP and TPC (2‐WAY ANOVA). LOG transformation used for TPC to normalize data.

### 2.2 Touch surface sampling and microbiological analyses

Microbiological sampling and microbiological analyses were described in more detailed elsewhere 26. In short, sampling was performed as a swap wiping method. Bacterial aerobic plate counts were analyzed using Trypticase Soy Agar plates using the mean value of parallel plates. Indicator bacteria occurrences (positive or negative finding) were confirmed from an enriched sample onto a selective plates, that is, Enterococcosel Agar‐plate (BD) (*Enterococci*), Mannitol Salt Agar plates (Labema Oy) (coagulase‐positive *Staphylococcus* and the presence of *Staphylococcus aureus* that are later in the manuscript referred as *S. aureus*) and MacConkey II Agar‐plate (BD) (*Enterobacteriaceae* and other Gram‐negative rods that are later in the manuscript referred to as Gram‐negatives). ATP as relative light units (RLU) was collected with a dry swab sample (Ultrasnap) and were read with the portable luminometer (Hygiena SystemSURE PLUS) following manufacturer's instructions (Hygiena International Ltd., Watford, UK). Total plate count (TPC) value ≥2.5 CFU cm^−2^ was used as surface hygiene benchmark as commonly used in hospitals 7, 27. In this study, we determined ATP ≥1 RLU cm^−2^ as an unacceptable (ie, dirty) guideline value based on 100 RLU limit 4, 27 that corresponds to limits 23 to 100 RLU/surface and thus is close to new guidelines of the manufacturer <25 for near‐patient areas and <50 for hospital public areas 28.

### 2.3 Optical measurements and equipment

Hyperspectral imaging system is described more detailed in our other study in a laboratory environment with *Escherichia coli* and *Bacillus subtilis* 24. In short, three color light source LEDs (red, green and blue [RGB]) were used as the excitation source. Digital Multiplex mode interface connected to computer and lighting networks, and enabled automatic changes of lights. Filter controller (ThorLabs Kurious) enabled only selected wavelengths from 420 to 720 nm to pass the filter. For each light, a continuous 20 nm increment was used in this study, resulting 48 raw images (as 1393 × 1040 pixels PNG images) per sample. JAI UV camera model CM‐140GE‐UV was used for imaging. Camera and light operated 40 to 80 cm away from the sample. Focus was adjusted for green light (wavelength λ = 525 nm) with camera using NiMaX interface. Data (images) collection was performed with LabVIEW program (version 2017). Operating parameters included only one light (RGB) with relative light intensities (I_rel_) from 50 to 255 while the other two lights were set to zero. Lower I_rel_ was chosen when the light source wavelength was close to filter wavelength to avoid overexposure.

### 2.4 Downstream clustering analysis of raw images

Microbial and dirt levels information were compared with the images of AutoDet to evaluate the cleanliness of the surface and to evaluate the real need for additional cleaning. Raw figures were cropped to smaller size figures with GIMP picture operating tool and Python (version 2.7.15) scripts for further analyses. Python NumPy and PyLab programs were used in cropping and visualization. The occurrence of stains was confirmed by comparing dirty and cleaned images that were taken in exactly same conditions. Transforming the figures to two‐colored images by a chosen color intensity threshold value and comparing to a cleaned figure enabled to spot also the human eye invisible stains. For the clustering analysis 24, cropped figures (0.5 × 0.5 mm) of dirty stain area (stain) and area next to the stain (background) as well as cleaned stain and background areas (cleaned surface area) were analyzed. In the clustering analysis, the intensity of two different sample conditions were transformed to color intensity vectors that were plotted to by two‐dimensional plot x‐ and y‐axes with the background. In principle, in the clustering plot, the samples with stains will cluster away from background samples. The clustering analysis for one selected sample (human eye invisible stain in a chair armrest) was performed manually by selecting only two different conditions (excitation light and bandpass filter). When utilizing all data (RGB, filters 420‐720 nm) with computer‐aided algorithms, images were analyzed using a k‐means 29. Support vector machine (SVM) classificator 30 was used to classify dirty and clean table surfaces.

### 2.5 Statistical analysis

Two‐way analysis of variance (2‐way ANOVA) (significance level *P* = .05) was used to detect differences between material (traditional and antimicrobial) and the sampling location for ATP and TPC (Table 1). Post hoc tests (Tukey honestly significant difference) were used to test differences within the sampling location. Logarithmic transformation was used for TPC statistical analyses in order to normalize the distribution in data, and half of the detection limit value was used in TPC with low growth. Normal distribution of the data was tested with Levene's test. Statistical analyses were performed using the Statistical Package for the Social Sciences (SPSS) for WINDOWS ver. 25.0 (SPSS Inc., Chicago, Illinois).

## 3 RESULTS

### 3.1 Real‐life hospital environment

Public hospital, including emergency lounge area and toilet (Figure 1A), was chosen as the environment for a real‐life case to study whether the designed optical AutoDet device (Figure 1B) functions also in the real‐life environment as was found efficient in the laboratory environment 24. The results suggest that the sampling location affected significantly (*P* < .05) for culturable bacteria count and ATP levels (Table 1) in studied surfaces. In addition, *S. aureus* seemed more abundant in toilets vs common areas. From 61 surfaces that were inspected for visual dirt, 23 surfaces indicated visual stains by the human eye. These visually dirty surfaces however did not show on average higher culturable bacteria counts (5 ± 8 CFU cm^−2^) than visually clean surfaces (7 ± 12 CFU cm^−2^, N = 38). Same trend was observed in ATP (1 ± 1 vs 4 ± 4 RLU cm^−2^, respectively). Most of the human eye visible dirt were detected from table surfaces (N = 19) that represented cleanest locations of the study (Table 1). Only three of the hospital surfaces were considered as very dirty by heavy growth, that is, 40 to 100 CFU cm^−2^ 27 including two toilet door lock knobs (51 and 191 CFU cm^−2^) and a chair armrest (85 CFU cm^−2^). Despite the lack of very dirty surfaces, 52% of culturable bacteria and 59% ATP samples exceeded hygiene guideline levels for common and near‐patient areas used in this study.

### 3.2 Detecting human eye invisible stain by manual image processing with two different wavelengths and bandpass filters

**Figure 2:**
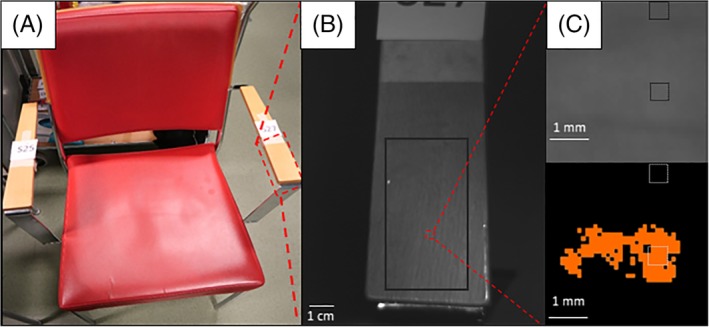
(A) A photograph of a chair with wooden varnished armrest from a real‐life hospital emergency area. (B) Raw figure of the armrest before cleaning using a green light (wavelength λ = ca. 520 nm, relative intensity I_rel_ = 50) and bandpass filter (λ = 500 nm) from which cropped figure (black rectangle) was used for further analyses. Dashed red square inside a cropped area highlights the location of the invisible stain (approximately 3 mm length, 1.5 mm height). (C) Magnification of the stain area as a raw figure (upper figure) and as modified image (lower figure) with 90° rotation of images to right. In the lower figure, an algorithm was used to visualize the invisible stain utilizing a threshold value 0.35 (intensity < threshold = black color, intensity > threshold = orange color). Dashed lines indicate stain and background locations for the clustering analysis

Instead of detecting the human eye visible stains (data not shown), more importance was given to the invisible stains. The optical device (AutoDet) was able to detect the human eye invisible dirt from a normal wooden armrest of a chair that is presented as a case study example (Figure 2A). ATP value of 1.9 RLU cm^−2^ (109 RLU/surface) confirmed the surface as dirty. Raw figures in any of the studied RGB wavelengths and filters (420‐720 nm) did not indicate the occurrence of the human eye invisible stain, for example, green light and filter 520 nm (Figure 2B). Using an algorithm with a certain threshold level and comparing the images of dirty and cleaned surfaces of the same circumstances, the stain and its location was revealed. Magnification of the raw image (Figure 2C) does not indicate the occurrence of the stain in top figure whereas the image where the threshold algorithm was utilized clearly reveals the stain as shown in the lower figure.

**Figure 3:**
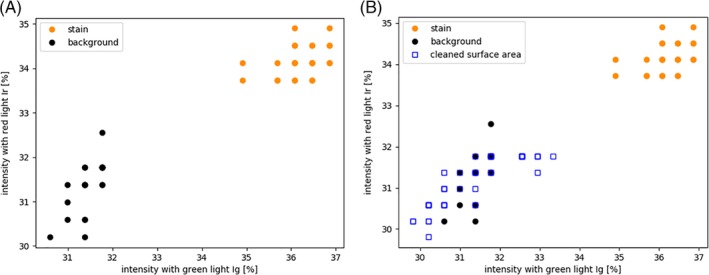
Clustering analysis with green light (excitation wavelength λ = 525 nm, relative intensity I_rel_ = 50) and red light (excitation wavelength λ = 625 nm, relative intensity I_rel_ = 255), both filtered by a bandpass filter (λ = 500 nm). Clustering analysis of (A) the dirty surface areas as shown in Figure 2C where the human eye invisible stain area (orange color) clusters away from the background area (black color) and (B) corresponding clustering analysis including data after cleaning

The phenomenon of different behaviors of two wavelengths was utilized in the manually performed clustering analysis, where an image was produced by selecting two areas (dashed squares in Figure 2C) and two excitation wavelengths (green and red light). The I_rel_ were plotted and the stain and the background separates from each other (Figure 3A). The orange area most likely represents organic dirt, and black area represents clean surface. This was confirmed by the comparison to cleaned surface areas where the cleaned areas (blue color) cluster together with background area (black color) and receive similar intensity values to the clean area before the cleaning (Figure 3B). The clustering analysis was able to differentiate the invisible stain manually with utilizing only two different operational conditions (red/green light, same bandpass filter). The surface was confirmed as clean based on low ATP value of 0.2 RLU cm^−2^ after cleaning.

### 3.3 Detecting human eye invisible stain by artificial intelligence methods utilizing all wavelengths and filters

**Figure 4:**
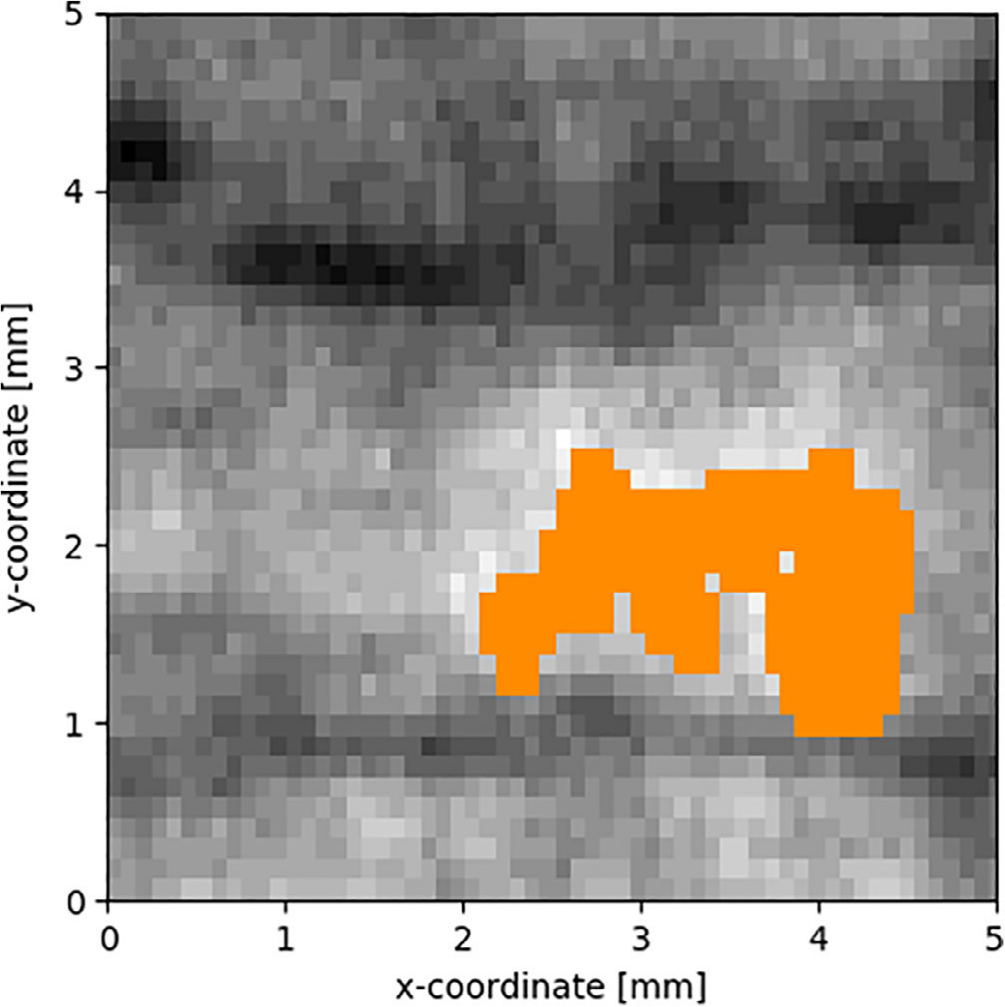
K‐means algorithm identifies the stain as shown in Figure 2C automatically as a continuation to the manual clustering method as shown in Figure 3. The stain is highlighted as orange color, and background image is performed with green excitation light and 500 nm filter. The method uses all data for the dirty surface, that is, red, green and blue excitation lights and filters 420 to 720 nm with 20 nm increments

The same example chair invisible stain was analyzed by computer‐aided parameters that included all three excitation lights (RGB) and filters (420‐720 nm) with 20 nm increments. The same invisible stain was identified by automatic K‐means algorithm (Figure 4) that used all the dirty data instead a threshold picked by hand (Figure 3). For k‐means, the raw image with green light and 500 nm filter was chosen as the background. Similar to manual method, the stain was revealed as shown as orange pixels in the same region representing the stain. This shows that if the location of the stain is known, it is possible to cluster she stain together automatically by utilizing all wavelengths. Based on manual and automatic clustering analyses presented above, the stain appeared as small clusters. Hence, the region of interest was divided to small squares and the SVM classified them as dirty or clean. Comparison to ATP values showed that in the clean surface, all the little squares classify as clean, but in the dirty case, both clean and dirty squares occur as the stains are not evenly distributed (Appendix S1).

## 4 DISCUSSION

This study further confirmed that the visual inspection of dirt is not enough to indicate whether the touch surface hygiene level is unsatisfactory as found earlier 7, 8 despite the visual inspection was not perfectly comprehensive due to varying shapes and colors of the touch surface materials. In this study, most of the human eye visible dirt was detected from relatively clean table surfaces probably by its best light reflecting properties. The hygiene levels in the studied touch surfaces were same order of magnitude for other hospital environments despite the typical variation in hospital surfaces 8, 31, 32. In this study, only few samples were considered as heavily contaminated by bacteria whereas many samples exceeded the current guideline values. Sampling location expectedly affected bacterial counts 26, 32, and especially high values were detected in toilet door lock knobs and busy registration desk chair armrests. Maintaining low bacteria counts is of high importance for patient safety as lower loads of bacteria is suggested to result in a lower risk for disease acquisition in a hospital environment 33.

In this study, we focused on the human eye invisible stains that pose challenges in the environmental surfaces, for example, in the generally clean health‐care units with frequent cleaning practices. We were able to detect successfully the human eye invisible stain from a dirty chair armrest by optical measurements where ATP value over 100 RLU confirmed the surface as dirty. The invisible stain was revealed manually by using only two visible light sources (green and red) and one filter, and the same result was confirmed utilizing automatically all three lights (RGB) and different filters between 420 and 720 nm. We were not able to confirm that the human eye invisible dirt was the cause of organic dirt detected on that surface (as seen as high ATP value) but it seems highly likely as a stain can appear in small clusters or nonevenly spread areas. For the analyzing of the dirt, the local occurrence of the stain instead of evenly distributing to the surface causes challenges for the algorithm development. With SVM, a correlation was found between ATP and our cleanliness estimator. Based on the optical imaging data acquired here, the dirt could not be identified. However, based on the properties of the stain emitting light at wavelength near 500 nm with lower excitation wavelengths, that is, green light as presented detailed and also with blue light (data not shown), riboflavin 18, 34 containing stain is a strong candidate. In that case, the stain could be a cell cluster or riboflavin rich food product.

In general, the optical inspection methods covering large areas aim at the same hygiene level as current techniques. The studied hospital was challenging environment due to high cleanliness typical for hospitals. If the optical method functions in a highly clean environment, it can be expected to function also in dirtier environments such as public places with less frequent cleaning. Methods like cultivation and ATP are still needed to indicate the cleanliness of a surface. Utilizing visible light is feasible in real‐life environments such as a hospital unlike harmful low wavelength UV lights. Thus, the optical methods utilizing visible wavelengths instead of UV‐light make a promising method for future cleanliness measurements in human occupied real‐life environments.

## CONFLICT OF INTEREST

The exact details of the algorithm in Supporting Information are proprietary information due to the commercial aspects of the Autodet project.

## Supporting information


**Appendix S1:** Supporting Information

Figure
