# Supplementary material for: Contamination detection by optical measurements in a real‐life environment: A hospital case study
Source: J Biophotonics. 2019 Nov 6;13(1):e201960069. doi: 10.1002/jbio.201960069 (PMC7065611; doi:10.1002/jbio.201960069)
Supplement: Supplementary file 1 — Appendix S1: Supporting Information [file JBIO-13-e201960069-s002.docx]

***Appendix S1 Supporting Information***

## Automated support vector machine method to classify dirty and clean surfaces

For additional step, a fully automated method using support vector machine was used to detect surface cleanliness on table surfaces. Based on manual and automatic clustering analyses in the manuscript, the stain appeared as small clusters. Hence, the region of interest was divided to small squares and the support vector machine (SVM) [1] classified them as dirty or clean based on the average and standard deviation of the intensity in the square. Counting the ratio of dirty and clean squares, an estimate for the cleanliness level was obtained. The exact details of the algorithm are proprietary information due to the commercial aspects of the Autodet project.

Figure 1 shows the correlation between the ATP measurement and the cleanliness estimate (hygiene index H). Each dot is one measurement of a 100cm^2^ area of a table, before (orange) and after (black) cleaning. There is a correlation how many squares are classified as dirty and ATP. All the little squares are clean in the clean case, but in the dirty case both clean and dirty squares occur as the stains are not evenly distributed. The automated algorithm can separate the clean surface from dirty one.


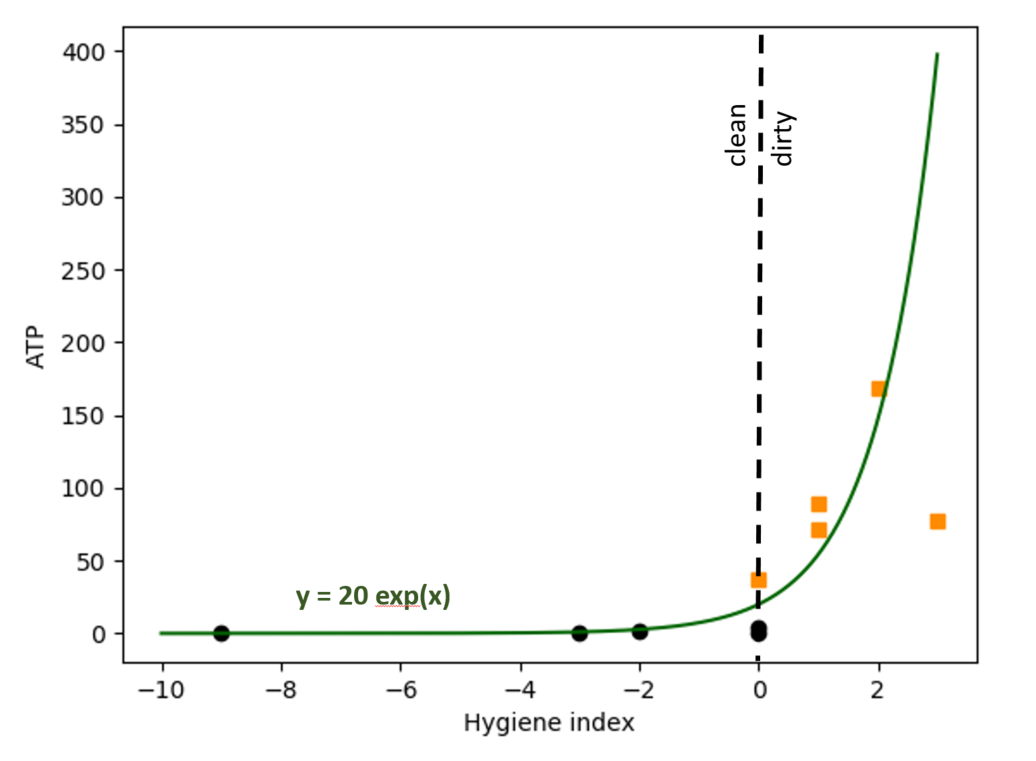


**Figure 1.** Correlation between the ATP measurement and the cleanliness estimate by the hygiene index. Orange dots present dirty surfaces and black dots cleaned surfaces. The hygiene index is based on machine learning classification of dirty and clean surfaces.

References:

[1] C. Cortes, V. Vapnik, *Mach. Learn.*, 1995, **20**, 273.
