# Supplementary figures and images for: Contamination detection by optical measurements in a real‐life environment: A hospital case study
Source: J Biophotonics. 2019 Nov 6;13(1):e201960069. doi: 10.1002/jbio.201960069 (PMC7065611; doi:10.1002/jbio.201960069)

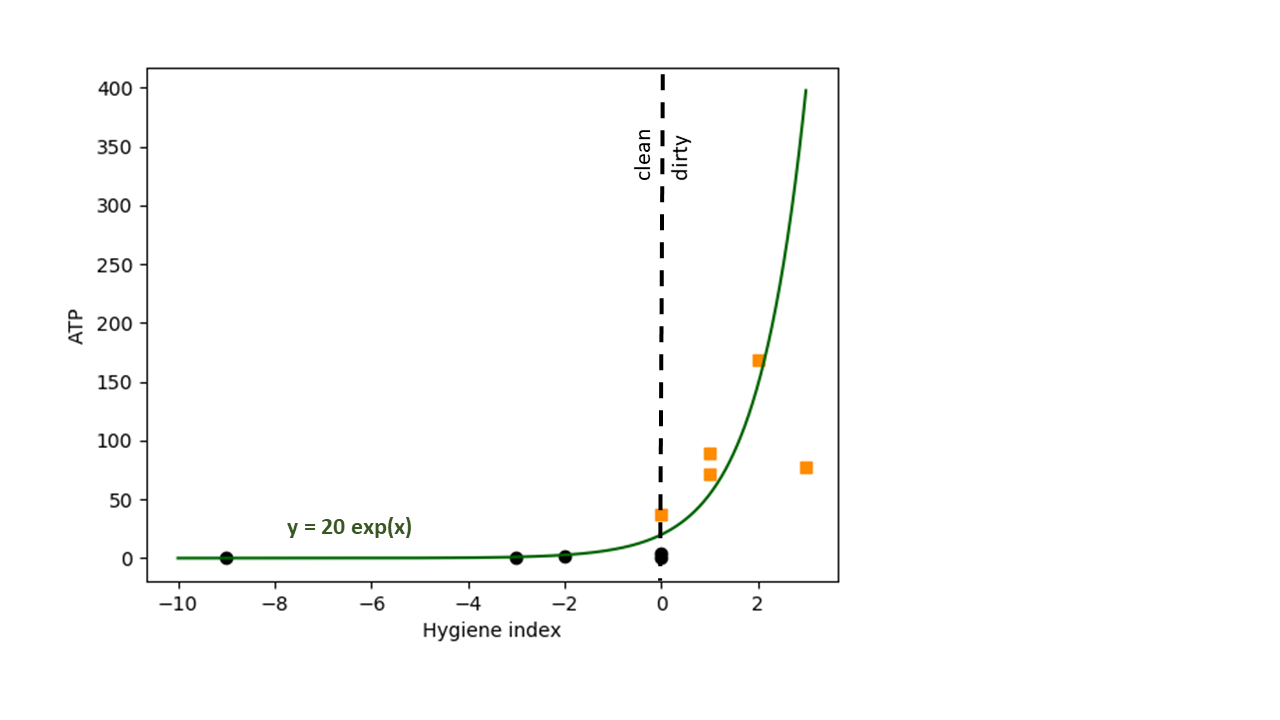

Supplement: Supplementary file 2 — Figure [file JBIO-13-e201960069-s001.tif]
